# Obesity and obesogenic growth are both highly heritable and modified by diet in a nonhuman primate model, the African green monkey (*Chlorocebus aethiops sabaeus*)

**DOI:** 10.1038/ijo.2017.301

**Published:** 2018-02-13

**Authors:** C A Schmitt, S K Service, A J Jasinska, T D Dyer, M J Jorgensen, R M Cantor, G M Weinstock, J Blangero, J R Kaplan, N B Freimer

**Affiliations:** 1 Department of Anthropology, Boston University, Boston, MA, USA; 2 Center for Neurobehavioral Genetics, The Jane and Terry Semel Institute for Neuroscience and Human Behavior, University of California—Los Angeles, Los Angeles, CA, USA; 3 Institute of Bioorganic Chemistry, Polish Academy of Sciences, Poznan, Poland; 4 South Texas Diabetes and Obesity Institute, University of Texas Rio Grande Valley School of Medicine, Brownsville, TX, USA; 5 Department of Pathology, Section on Comparative Medicine, Wake Forest School of Medicine, Winston-Salem, NC, USA; 6 Department of Human Genetics, University of California—Los Angeles, Los Angeles, CA, USA; 7 The Jackson Laboratory for Genomic Medicine, Farmington, CT, USA

## Abstract

**Objective:**

In humans, the ontogeny of obesity throughout the life course and the genetics underlying it has been historically difficult to study. We compared, in a non-human primate model, the lifelong growth trajectories of obese and non-obese adults to assess the heritability of and map potential genomic regions implicated in growth and obesity.

**Study population:**

A total of 905 African green monkeys, or vervets (*Chlorocebus aethiops sabaeus*) (472 females, 433 males) from a pedigreed captive colony.

**Methods:**

We measured fasted body weight (BW), crown-to-rump length (CRL), body-mass index (BMI) and waist circumference (WC) from 2000 to 2015. We used a longitudinal clustering algorithm to detect obesogenic growth, and logistic growth curves implemented in nonlinear mixed effects models to estimate three growth parameters. We used maximum likelihood variance decomposition methods to estimate the genetic contributions to obesity-related traits and growth parameters, including a test for the effects of a calorie-restricted dietary intervention. We used multipoint linkage analysis to map implicated genomic regions.

**Results:**

All measurements were significantly influenced by sex, and with the exception of WC, also influenced by maternal and post-natal diet. Chronic obesity outcomes were significantly associated with a pattern of extended growth duration with slow growth rates for BW. After accounting for environmental influences, all measurements were found to have a significant genetic component to variability. Linkage analysis revealed several regions suggested to be linked to obesity-related traits that are also implicated in human obesity and metabolic disorders.

**Conclusions:**

As in humans, growth patterns in vervets have a significant impact on adult obesity and are largely under genetic control with some evidence for maternal and dietary programming. These results largely mirror findings from human research, but reflect shorter developmental periods, suggesting that the vervet offers a strong genetic model for elucidating the ontogeny of human obesity.

## Introduction

The mechanisms by which longitudinal and developmental processes contribute to risk for adult obesity outcomes remain largely unclear, but available evidence suggests the importance of critical periods during which high or low caloric intake, body weight (BW) and weight gain particularly influence adult obesity.^1^ Specific early childhood growth patterns, such as rapid rate or acceleration of growth in BW, are among the strongest predictors of adult obesity.^1, 2, 3^ Most such evidence is necessarily derived from either cross-sectional studies or comparisons of intra-individual differences in a few clinical time points, rather than consistent monitoring of obesity-related traits across the lifespan.

The genomic factors underlying obesity risk have become more clear, although much remains to be learned. Obesity is highly heritable (*h*^2^ of ~40–70%), and human genome-wide association studies (GWAS) have identified dozens of replicable loci showing associations with obesity-related phenotypes,^4, 5, 6, 7^ although these loci do not explain the majority of the heritability for these traits.^7^ Some of these loci appear to act in an age-dependent manner, suggesting that the ontogeny of obesity is both genetically dynamic and pleiotropic.^8, 9, 10, 11^ Relatively few projects have sought to both characterize long-term ontogenetic patterns associated with adult obesity while also examining the potential genetic basis of such patterns.^11^

The dearth of such research in humans may be due to the lack of appropriately large longitudinal samples and the difficulty of controlling for confounding dietary and environmental variables over long developmental periods.^12, 13^ While rodent models have helped elucidate the phenotypic impact of human genes related to obesity, their physiologic divergence from humans may limit their utility.^14, 15^ Non-human primates (NHP) are phylogenetically more closely related to humans, share more similar genomic structure, and are more appropriate physiological models for human obesity and fat deposition than rodents.^14, 15, 16, 17^ As in humans, obesity occurs in captive NHPs even with diets low in total and saturated fats and simple carbohydrates.^16, 17, 18, 19, 20, 21^ Perhaps most importantly, NHPs also exhibit growth trajectories more similar to those of humans^22^ than rodents, yet develop and reproduce comparatively quickly, allowing collection over short periods of time of large developmental samples.

We therefore utilized extensive longitudinal measures (dating from 2000 to 2015) from a pedigreed colony of African green monkeys, or vervets (*Chlorocebus aethiops sabaeus*), an established NHP model for human obesity,^21, 23, 24, 25, 26, 27^ to assess the heritability of adult obesity phenotypes; to characterize and differentiate developmental patterns between those individuals that become obese as adults from those that do not; to assess specific growth patterns for evidence of both heritability and environmental programming; and to use multipoint linkage analysis to identify regions of the vervet genome associated with these traits.

## Methods

### Study population

The Vervet Research Colony (VRC) is a multigenerational pedigreed colony (housed at UCLA until February 2008 and at the Wake Forest Primate Center since that time) that currently consists of ~300 vervets. Details regarding vervet physiology with reference to obesity, development, colony maintenance and housing conditions are reported elsewhere.^21, 23^ Adult male availability in the VRC is low because, in keeping with natural dispersal patterns, most males are removed as they near adulthood.

### Dietary considerations

The VRC vervets are typically fed a standard monkey chow diet, LabDiet 5038, Monkey Diet, here referred to as ‘Standard’ (caloric content: 69% carbohydrates, 18% plant protein, 13% fat, with 5% weight as crude fiber; Purina, St. Louis, MO). Through April and May 2004, the monkeys were fed a gradually increasing proportion of an intervention diet, or ‘ID’—LabDiet 5052, Fibre-Balanced Monkey Diet (caloric content: 58% carbohydrates, 27% plant protein, 15% fat, with 12.4% weight as crude fiber).^26, 27^ This gradual introduction was meant to ease them into 100% ID by June of 2004, after which they were exclusively fed ID until January/February 2008, when the diet of the colony was changed back to Standard chow (see Supplementary Figure 1 for a detailed timeline). *Ad libitum* access to food, water and opportunities for exercise were available to all animals throughout the study period. Animals were supplemented with enrichment foods, such as fruits and vegetables, 1–5 days per week.

Given that maternal diet during gestation is hypothesized to influence long-term offspring growth^28^ and has been implicated in long-term adipocyte programming in humans,^29^ we included it as a covariate in analyses. Given that monkeys selectively ate the standard chow when it was available, infants born up until May 2004, when proportion of ID surpassed 50%, are included in the Standard cohort. Although prenatal response to maternal diet has been hypothesized to vary based on trimester of exposure,^3^ we chose to define individuals as having gestated with the modified ID if their mother ate majority ID at any point during the 165-day gestation period. Certain critical windows of postnatal age are also hypothesized to influence the effect of dietary changes on subsequent growth,^3^ and so the timing in the shift to ID is examined using covariates indicating three postnatal periods at which majority ID was introduced: postnatal period 1 (PN1, from birth to 2 years old), postnatal period 2 (PN2, from 2 to 5 years old), and adulthood (from 5 years old).

Study protocols were approved by the University Institutional Animal Care and Use Committees of both UCLA and Wake Forest School of Medicine.

### Measurements

We collected clinical measures annually from 2000 to 2007 and thrice yearly from 2008 to 2015 to characterize body condition. Measurement sessions were facilitated by sedation with intramuscular ketamine (8–10 mg/kg). We measured BW (in kg) using an electronic scale, and waist circumference (WC, in cm) by placing a tape measure around the abdomen at the umbilicus. We measured crown-to-rump length (CRL, in cm), the equivalent to sitting height in anthropometric measures, from the crown to the bottom of the pubic bone using calipers or a stationary slide scale. CRL and BW were consistently taken from 2000 to the present, and interobserver reliability measures and training were conducted within both the UCLA and Wake Forest facilities, ensuring within-site consistency of measures. Although interobserver reliability measures were not explicitly taken across sites, post hoc comparisons of CRL measures taken at both UCLA and Wake Forest suggest reasonable consistency between sites for our analyses (Supplementary Figure 2). Measures of WC were only taken consistently from 2008 to the present, and so earlier measures were not used. Body-mass index (BMI) was calculated as the weight (in kg) divided by CRL (in m) squared. Due to longitudinal restrictions on matched BW and CRL measures, BMI is only reliably available from 2008 to the present.

### Genotype data

Genotype data were generated through whole-genome sequencing of 725 members of the VRC.^30^ Genotypes from 721 VRC vervets that passed all QC procedures can be directly queried via the EVA at EBI (www.ebi.ac.uk/eva). Analysis in this paper used the Linkage Mapping SNP Set, consisting of 147,967 markers on the 29 vervet autosomes. In this set of ~148K SNPs, there were an average of 58.2 SNPs per Mb of vervet sequence, and the average gap size between adjacent SNPs was 17.5 Kb.

We used the software package Loki,^31^ which implements Markov Chain Monte Carlo, to estimate the multipoint identical by decent (MIBD) allele sharing among all vervet family members from the genotype data at 1 centi-Morgan (cM) intervals. The correspondence between physical and genetic positions in the vervet was facilitated by a vervet linkage map,^32, 33^ constructed using a set of 360 STR markers. Both the physical and genetic position of these markers was known, and genetic locations of SNPs were found by interpolation.

### Analytical methods

#### Adult measurements

As in humans, we defined adult individuals of both sexes as obese if WC was in the upper 20th percentile of colony measures, or >40.5 cm.^21^ This ‘obese’ WC phenotype in the VRC carries with it a number of comorbidities associated with metabolic syndrome, such as hyperinsulinemia and increased blood triglyceride and insulin concentrations,^21^ and so we are confident that it excludes healthy animals that happen to be larger. We defined animals obese for three or more consecutive measures, excluding measures of pregnant females, as chronically obese. For adult measurements, we took the mean of all measures from age at stable adult size (in the VRC, 5 years of age onward)^34^ to account for natural fluctuations in weight.

#### Growth parameters

We modeled growth only for those individuals with at least 6 measures of BW. We used k-means longitudinal clustering on BW to define similar clusters of individual growth trajectories without predefined obesity status, implemented using the package *KmL*,^35^ version 2.3, in R,^36^ as individuals may show signs of obesogenic growth (or a growth pattern leading to an obese phenotype) without yet having become obese. This method is also robust to missing values in individual trajectories, and requires no assumptions regarding trajectory shape.^35^ We identified BW clusters only, as this variable had the largest sample size for a trait that reflects changes in individual adiposity over time. Each sex was assessed separately to accommodate sexual dimorphism in growth.^37, 38^ We used the Caliski & Harabatz criterion^35^ to define the optimal number of growth clusters. By implementing SOLAR^39^ to perform statistical comparisons of average adult traits between clusters, we used the colony pedigree structure to control for relatedness.

We modeled individual growth trajectories using nonlinear mixed effects (NLME) with a logistic growth curve defined as:


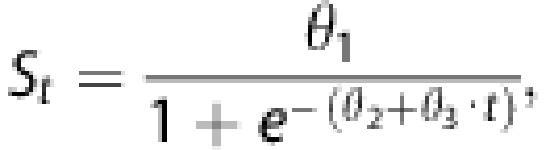


where *S*_*t*_, size at age *t*, is a function of three growth parameters. *θ*_1_, the asymptote of growth, represents eventual adult size. *θ*_2_ is an indicator of growth duration which, in combination with *θ*_3_ (using the relationship −*θ*_2_/*θ*_3_), represents the point at which 50% of adult size is reached in the logistic model. *θ*_3_, the growth rate constant, represents the rate of growth between zero and the asymptote. We fitted models using the function *nlmer* in package *lme4*^40^ version 1.1-7 in R, which allows for missing and unmatched data points between subjects, and for each curve parameter to be estimated simultaneously, ensuring a consistent sample size for each age.^41^ We modeled growth parameters as fixed effects. Random effects were included for growth parameters associated with subject ID to account for repeated measurements on the same subject. We constructed independent models for each KmL-assigned cluster within each sex. This approach allowed us to extract estimated growth parameters for each individual subject from the models using the random effects, while also permitting a comparison of mean growth trajectories for each predefined growth cluster within sex. The reduced frequency of measures for BMI and WC resulted in a reduction in sample size that prevented our modeling growth for these traits.

#### Quantitative genetic analysis

We assessed the narrow-sense heritability (*h*^2^) of average adult obesity traits and subject-specific model-estimated growth parameters, using a maximum likelihood variance decomposition method implemented in SOLAR.^39^ For adult traits, mean age was regressed out and residuals were used as the dependent variable. Covariates included sex, maternal diet during gestation, birth location (UCLA or Wake Forest), birth year, the three covariates representing the age at which ID was introduced, and interaction effects between those variables if they contributed significantly to trait variance. The reduced sample size for WC prohibited the exploration of full models, and so age, sex, ID exposure, the interaction of sex and ID exposure, and birth year were the only covariates included. All traits were inverse-normal transformed prior to analysis to correct for residual kurtosis. We modeled maternal effects (*c*^2^) in SOLAR by defining maternal identity as a household effect within the environmental component of variance.

#### Multipoint linkage analysis

We implemented whole-genome multipoint linkage analysis of heritable traits in SOLAR, which uses a variance components approach to partition the genetic covariance between relatives for each trait into locus-specific heritability (*h*^2^*q*) and residual genetic heritability (*h*^2^*r*). The null hypothesis of no linkage (*h*^2^*q*=0) was tested at 1 cM intervals, and the significance of the maximum likelihood estimate of *h*^2^*q* evaluated using the log_10_ ratio of the likelihood ratio (LOD score). In accordance with established standards,^42^ genome-wide statistically significant linkage was set at LOD ⩾ 3.3, and suggestive linkage at LOD ⩾ 1.9. Traits were adjusted for covariates age, birth year, birth location, sex, birth diet, interaction between sex and birth diet, indicators for HF exposure and interaction of these indicators with sex. The binary trait of chronic obesity was also analyzed, using a liability threshold model incorporated into SOLAR.

Linkage results were compared to the annotated physical map of the vervet reference genome assembly^43^ ChlSab1.1 (GCA_000409795.2) in Ensembl v. 83.1.^44^ Noted gene regions were then checked against annotations in both NCBI AceView^45^ and GeneCards^46^ to determine known functionality.

## Results

Based on our analytical criteria, of the 1665 individuals measured in the VRC from 2000 to 2015, a total of 905 were suitable to assess either adult obesity traits, growth patterns or both. A total of 626 (*n*_F_=395; *n*_M_=231) had both known parentage and adult measures, and so were used to assess adult obesity traits. The mean number of measures for females was 13.6, and 8.6 for males. Mean age, trait values, and obesity status for individuals can be seen in Table 1. Of these, 607 were born at UCLA, and 19 at Wake Forest. For individuals with adult measures, initial ID exposure occurred during gestation (*n*=80), PN1 (*n*=73), PN2 (*n*=140), or adulthood only (*n*=202). There were 29 birth years represented in the sample (1980–2009; Supplementary Figure 3). Household effects for adult traits included 268 mothers.

Growth patterns were modeled using a sample of 800 individuals (*n*_F_=433; *n*_M_=367). Of these, 750 were born at UCLA, and 50 at Wake Forest. Initial ID exposure occurred in gestation (*n*=171), PN1 (*n*=97), PN2 (*n*=166), or during adulthood (*n*=203). There were 29 birth years represented in the sample (1980–2009; Supplementary Figure 4). Household effects for growth traits included 304 mothers.

### K-means longitudinal clustering denoted one heavy and one light cluster in each sex; the heavy cluster is related to adult obesity

**Figure 1:**
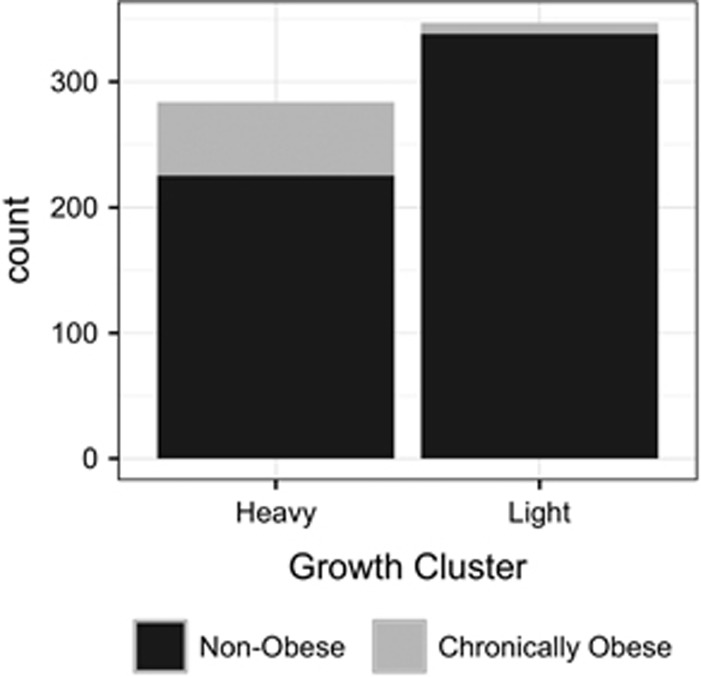
Chronic obesity outcome in adults assigned to heavy vs light growth clusters. Chronically obese individuals are defined as having a waist circumference above 40.5 cm for more than three consecutive measurements.

The KmL algorithm supported two growth clusters in each sex, one ‘heavy’ and one ‘light’. Adults in the heavier cluster showed significantly greater BW, as expected, but also larger WC, and higher BMI. CRL, a more explicitly skeletal measure, also showed significant differences between clusters for both sexes (Table 1; *P*<0.0001 for all trait differences between sex and cluster). For both sexes, obese individuals were more likely to be found in the heavy growth cluster (Males: *P*=0.002; Females: *P*<0.0001), suggesting that this cluster, or growth pattern, corresponds to clinically defined adult obesity outcomes (Figure 1).

### NLME accurately modeled the data, and showed marked differences in growth parameters between heavy and light clustered males and females for BW and CRL

**Figure 2:**
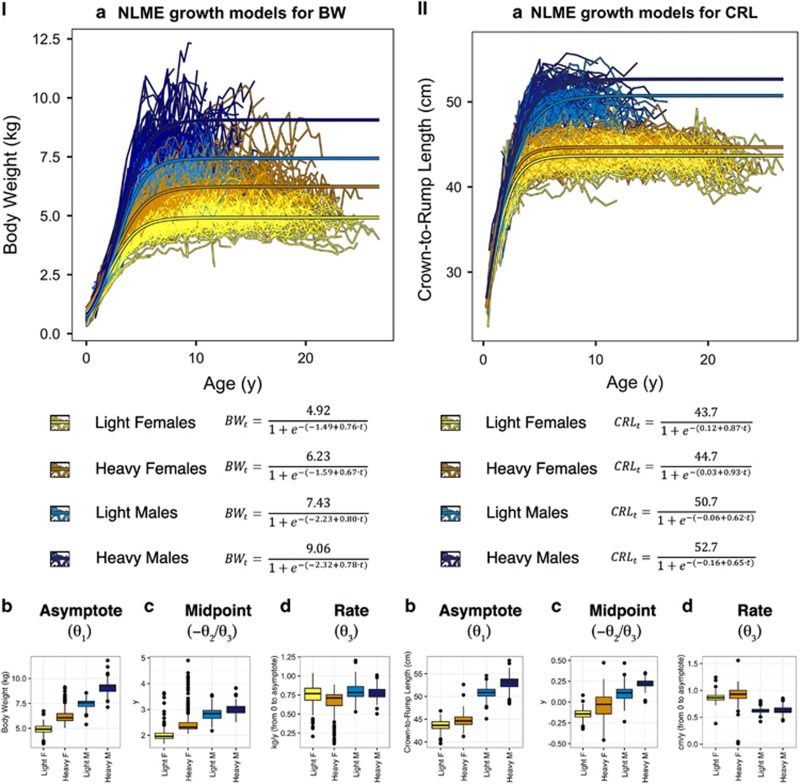
Measurements and NLME logistic growth model output for (I) body weight (BW) and (II) crown-to-rump length (CRL) in the VRC. Color-coding indicates sex and growth cluster assignment: dark blue=heavy males, light blue=light males, dark yellow=heavy females, light yellow=light females. In plot (**a**), each thin line connects individual measurement points for a single vervet, while thicker trend lines represent the average growth model for each sex/cluster. The boxplots show the mean and interquartile ranges of random effects deviations from the population average growth model, divided by cluster, for each growth parameter—(**b**) *θ*_1_, the asymptote of growth, measured in kg; (**c**) −*θ*_2_/*θ*_3_, the midpoint of growth, measured in years; and (**d**) *θ*_3_, the growth rate constant, measured in years^−1^—that describe individual NLME logistic growth models grouped by sex/cluster and color-coded using the same system as (**a**). Parameter values are derived by adding random effects of subject identity to the mean parameter values for each sex/cluster.

Models for BW growth displayed a large difference between the sexes, and also significant differences between growth clusters within sex (Figure 2Ia). As in adult measures, males overall reached a larger adult weight than females (Figure 2Ib, Table 1; *P*<0.0001) and attained this heavier weight by extended growth, or bimaturism, (Figure 2Ic) and with a slower rate of growth (Figure 2Id). Within both sexes, the heavy growth clusters had significantly higher BW than the light, and appeared to develop this higher BW by a similar form of bimaturism represented by a longer duration of growth (females, *P*=0.0005; males, *P*=0.0009) and a slower rate of growth (females, *P*<0.0001; males, *P*=0.009), compared to those in the light cluster.

Growth parameters also varied by sex and cluster for CRL (Figure 2IIa). While sexual dimorphism was seen between males and females, individuals assigned to the heavier growth cluster within the models became much larger for their sex than those assigned to the lighter growth cluster (Figure 2IIb; *P*<0.0001 in both sexes), and had more exaggerated bimaturism (Figure 2IIc; *P*<0.0001 in both sexes), but the heavy growth clusters appeared to have similar rates of growth compared to the members of their sex assigned to the lighter cluster (Figure 2IId).

Pearson correlations between observed and predicted values for both BW and CRL were high during development (BW: *r*>0.98; CRL: *r*>0.97), and throughout the life course (BW: *r*>0.79; CRL: *r*>0.70) indicating that these models accurately fit the observed patterns of individual growth.

### Important environmental covariates include sex and the switch to the ID diet

Sex was a significant covariate for all traits with the notable exception of chronic obesity (Table 2). Maternal identity, used here as an indicator for maternal effects (*c*^2^), did not explain environmental variance in average adult measures except for a very small proportion of variance in BMI and WC, and did not explain any environmental variance in growth parameters (Table 2).

**Figure 3:**
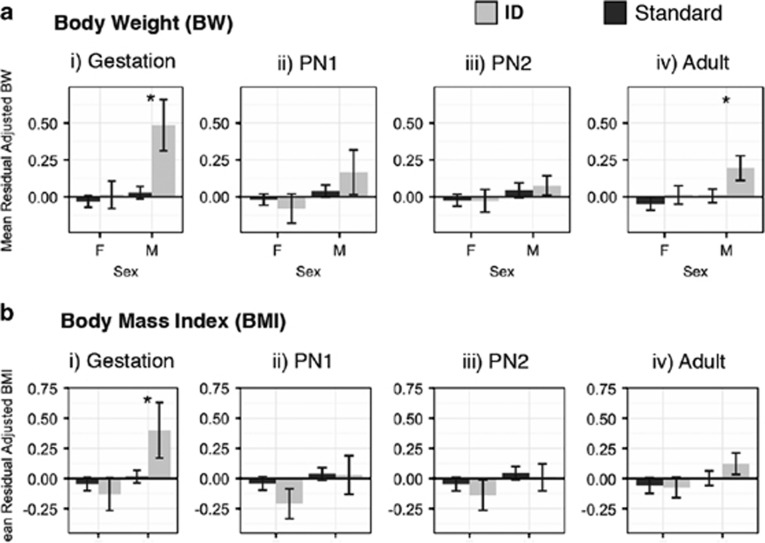
Mean and standard error of residual differences by sex in age-adjusted (**a**) BW and (**b**) BMI for individuals fed a Standard diet (in black) and those who experienced a shift to ID (in gray) during (i) gestation (maternal shift to HF while gestating that individual), (ii) during the first 2 years after birth (PN1), (iii) during the subsequent 3 years after birth (PN2), and (iv) during adulthood. Residual values were attained after regressing out significant covariates, here including age and growth cluster assignment.

Dietary covariates had a complex relationship to most adult and growth measures. Maternal diet during gestation was a significant covariate for CRL, with a similar effect in males and females (Supplementary Figure 5). The interaction between sex and maternal diet during gestation was a significant covariate for BW and BMI, suggesting sex-specific effects of maternal diet on body condition (Table 2). Indeed, for both BW and BMI, males gestated while their mothers ate ID were markedly heavier or larger than those whose mothers ate Standard (Figures 3a and b).

While switching to ID at any age during development (PN1, PN2) had a significant effect on adult CRL, only switching during PN1 was a significant covariate for BW. Switching to ID during adulthood was a significant covariate for BMI, and the effect was dependent on sex (Figure 3). In contrast, the analysis of WC did not detect any dietary effect, although the sample size for WC was very low compared to other traits (Table 2, Supplementary Figure 6).

The switch to ID also had a significant effect on most growth parameters for both traits, although, the pattern was not consistent and was often mediated by sex (Table 2; Supplementary Figures 7–12). The interaction between sex and maternal diet during gestation was significant for all growth parameters for BW and for two parameters of CRL (*θ*_1_ and *θ*_3_), as were the interactions between sex and postnatal developmental periods PN1 (BW and CRL *θ*_1_), PN2 (BW and CRL *θ*_1_ and *θ*_2_) and adulthood (BW *θ*_2_ and *θ*_3_). Although there are significant interactions with sex for the influence of all dietary covariates on growth parameters, how the influence of diet differs by sex is unclear from model residuals.

### All measurements had a significant genetic component, and multipoint linkage analysis showed several genome-wide suggestive, but not significant, loci containing genes implicated in human metabolic disorders

All average adult measures of obesity-related traits, including chronic obesity, were both highly and significantly heritable (*h*^2^). Growth traits were also significantly heritable, although estimates of heritability were not uniformly high (Table 2).

**Figure 4:**
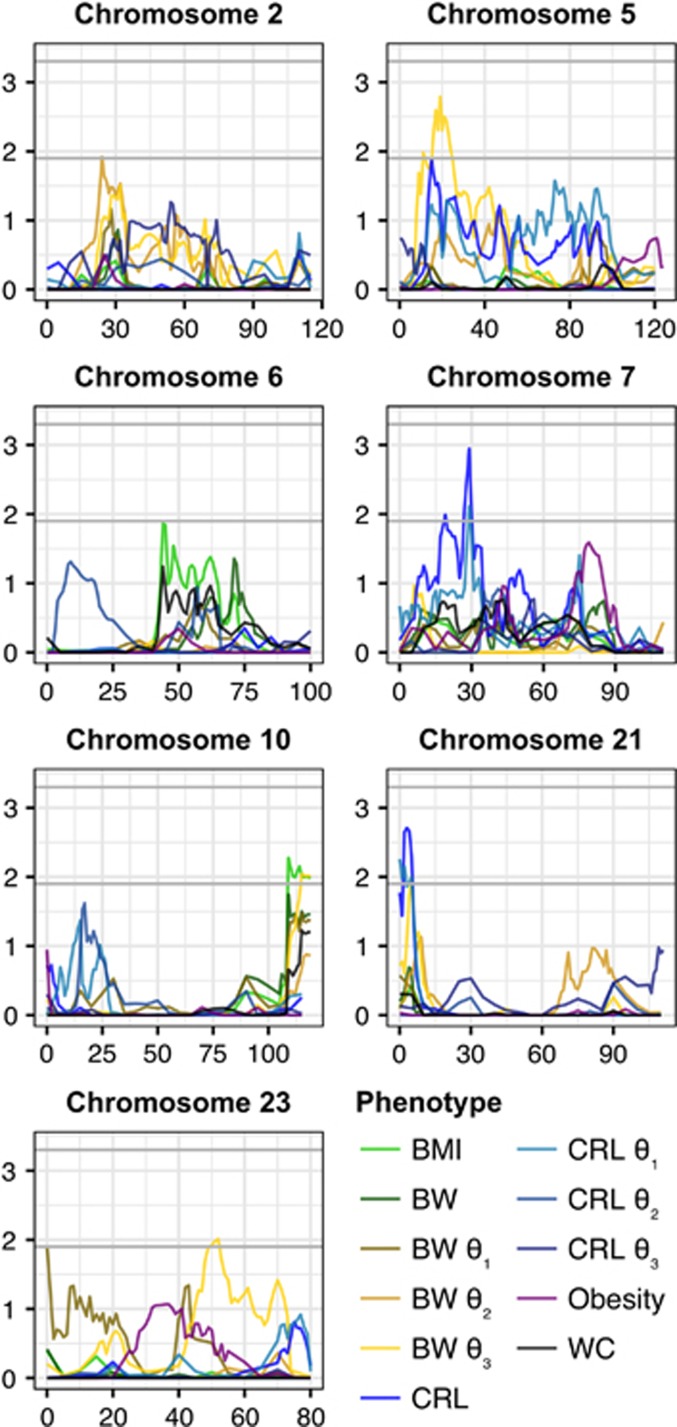
Results of the multipoint linkage analysis for all traits across the vervet genome (autosomal chromosomes with suggestive results only). Each gray horizontal line represents genome-wide suggestive (LOD ⩾ 1.9) and genome-wide significant (LOD ⩾ 3.3) levels.

Despite having a significant genetic component to phenotypic variability, no traits achieved genome-wide statistically significant linkage. We did identify seven regions with suggestive linkage for BMI, CRL, all three growth parameters for BW, and the parameter representing asymptote of adult growth for CRL (Table 3, Supplementary Table 1; Figure 4).

## Discussion

Vervet monkeys show two distinct growth patterns, one of which corresponds to adult-onset obesity. The association of the heavier growth pattern with obese adult outcomes implies that this pattern is an important but not necessary component of adult-onset obesity, as some individuals from the light cluster also became obese. As in humans, obesity-related trait states and the patterns of growth leading to those traits appear to be largely under genetic control, but with evidence that diet can have a significant impact on both adult state and on growth rates during ontogeny, especially when diet is shifted *in utero*. Maternal effects, as we measured them, appeared to be largely absent outside of maternal dietary covariates, which had a significant effect on almost all traits related to body condition and CRL, as well as on most parameters of growth.

There is a widely recognized utility in using extremes in clinical phenotypes to assess the genetics underlying a trait.^47^ However, by using an agnostic clustering method to define underlying growth patterns leading toward a heavier adult size associated with obesity outcomes, we identified a greatly expanded number of individuals sharing an obesogenic phenotype, the majority of which were not yet obese by standard clinical indicators. These results imply that variation in lifelong growth patterns may indicate a common etiology underlying adult obesity that would have been missed based only on extreme values of static clinical indicators. By averaging longitudinal data to define adult obesity-related measures, we have also been able to detect higher additive genetic heritability for obesity-related traits in this taxon, as compared to earlier studies.^21^

The significant impact of maternal diet during gestation on both obesity-related adult body condition and growth patterns is intriguing, and is an effect that has also been noted in humans.^1, 48, 49^ Although this result may be an artifact of the relatively reduced sample size available for adult males in the VRC, if validated in a larger sample it may reflect evolutionary strategies related to energetic investment and regulation of offspring growth. The vervet response to ID suggests nutritional stress: mothers lost ~10% BW and subsequently altered their behavior by limiting access to the nipple and increasingly rejecting infants.^26^ It is possible that this led to low birthweight, data unfortunately lacking in this data set, followed by subsequent catch-up growth in offspring—represented here by longer growth trajectories—resulting in the over-storage of energy in the form of obesity.^50, 51^ Subsequent work, such as that by Wells,^49, 52^ however, elaborates upon the hypothesized physiological mechanisms underlying maternal programming leading to adult obesity to include many other intervening factors throughout development. Overall, this ‘thrifty phenotype’ hypothesis posits that mechanisms of phenotypic plasticity—such as variation in gene expression due to maternally mediated epigenetic programming—have evolved to alter offspring development in preparation for postnatal nutritional stress in humans. That these mechanisms may also be present in a model like vervets is promising for the future discovery of these mechanisms.

The sex-specific component of this developmental effect may also benefit from an evolutionary perspective. Although maternal rejection of offspring in the VRC while eating ID occurred equally across sexes, it is possible that males are especially susceptible to nutritional stress *in utero* and while nursing.^53^ Classic theoretical work in evolutionary biology does stipulate that mothers in female philopatric species (like vervets, where sons disperse and daughters remain in their birth group) should invest preferentially in daughters if resources are scarce (as daughters may potentially out-reproduce sons under such conditions).^54^ It has already been posited that this outcome may be, in part, mediated by fetal or lactational programming favoring robust daughters over sons,^55^ although there are now several competing evolutionary hypotheses that may explain sex-specific effects in maternal programming.^56^ These results may explicitly link the mechanisms of obesogenic growth to this phenomenon of natural selection. To follow this line of inquiry further: (1) more males must be sampled to rule out statistical anomaly; (2) other post-natal interventions by which mothers may introduce sex bias in offspring development, such as lactational programming^57^ and possibly sex-biased behavioral interventions^26, 58^ must be controlled; and (3) patterns of gene expression in fatty tissues or epigenetic modifications surrounding candidate genomic regions should show consistent differences between offspring exposed to different maternal dietary interventions *in utero* and/or when nursing.

Taken together, these results suggest the joint influence of genetic predisposition and dietary effects throughout the lifespan leading to adult obesity outcomes. That this relationship is yet somewhat unclear in this analysis calls for further investigation. That BW, BMI and CRL are influenced by dietary covariates during gestation (Figure 4; Supplementary Figure 5) while WC (Supplementary Figure 6) is not and only CRL is consistently influenced throughout development by diet (Supplementary Figure 5), suggests certain developmental periods and pathways are more influenced by maternal and environmental programming than others, leading to mosaic patterns of altered development and adult phenotypes.^59^ The full scope of this signal may not be clear in our sample due to the coarse longitudinal grain of the data and limited sample size for some traits, like WC. More direct phenotyping of obesity traits, such as assessments of adiposity via dual emission X-ray absorptiometry, could also yield more informative results.

The nature of this study leaves little room to speculate which component of the ID may be affecting obesity and growth outcomes. Although nutritional stress appeared to occur in the colony when eating ID,^26^ in the absence of experimental dietary controls it is unclear whether the increased fiber intake, or altered ratio of proteins to carbohydrates might have been responsible. Increased fiber intake is associated with weight loss in humans, although the mechanism is still under debate.^60^ A higher ratio of protein to carbohydrates alone is not associated with weight loss in human clinical trials,^61^ but may be if it also results in reduced intake, which may have been the case in the VRC. Similarly, it is debatable whether increased protein intake alone could be responsible for the weight and growth changes seen. There is no evidence that the monkeys were protein deficient on the Standard diet, and evidence is mixed regarding whether protein in excess of recommended levels without concomitant increases in intake of other micronutrients significantly alters weight or musculature.^62^ Although *in utero* high-protein diets in rats can cause higher body weight and fat deposition in adults, this only occurs in females rather than males,^63, 64^ suggesting a dissimilar etiology to the process observed here in vervets if high protein is the responsible dietary factor.

Our inability to identify a genomic location with significant linkage to any trait suggests that, as in humans, these phenotypes are highly polygenic, with multiple loci each accounting for a small fraction of the estimated heritability.^65^ Simulations in SOLAR indicate that with ~600 phenotyped individuals in the vervet pedigree and trait heritability of 65%, we have ~90% power to detect a LOD of 3.3 if the locus-specific heritability is 17% or greater, suggesting that individual loci contributing to these traits likely have smaller effects. Those loci with suggestive linkage are compelling: 48 of the protein coding sequences in the 7 vervet linkage regions with LOD >1.9 are associated with diseases or cellular functions linked to metabolic disorder, growth disorders, or obesity in humans. These include the type 2 diabetes susceptibility region of chromosome 20, several other loci associated with insulin dysregulation and type 1 diabetes, lipid metabolism, atherosclerosis and coronary artery disease, thyroid disorders and many loci in the TNF-α/NF-κB pathway specifically associated with obesity in vervets^24, 25^ (Table 3; see Supplementary Table 1 for more detail and references). That some of these loci are linked exclusively to growth traits rather than static adult traits suggests that breaking obesity phenotypes down into constituent parts (such as elements of growth that contribute to adult phenotypes) may be a way of narrowing the variation contributing to complex phenotypes into more statistically detectable units. In the future, incorporation of growth cohorts beyond 2015 will increase power to detect linkage regions associated with each trait, and fine mapping of candidate loci within linkage regions—and a closer investigation of SNP variation in association with these traits—will better illuminate their potential role in obesity and obesogenic growth in vervet monkeys.

Ultimately, these results reveal an intriguing new model for the genomics and development of adult-onset obesity that can take into account genetic predisposition, growth and dietary influences on adult obesity. While existing NHP models are already addressing a number of questions related to obesity and development (e.g., *Macaca*,^18, 19^ *Papio*^20^ and *Callithrix*^66, 67^), the vervet model is unique in its opportunities for comprehensively integrating translational research on the ontogeny and genomic etiology of obesity in captive populations (as presented here) with investigations of these phenotypes in extensive wild samples from a similar genetic background.^14^ Such integration will open the door to understanding not just the proximate mechanisms by which individuals become obese, but may also address the adaptive framework by which these mechanisms evolved.

## Figures and Tables

**Table 1:** Summary statistics for average adult body condition values

	*N*	*AGE*	*Obese Chronic*	*BW*	*CRL*	*WC*	*BMI*
*Females*	*395*		*58*				
Heavy cluster	172	10.1 (3.93)	49	5.93 (0.63)	44.6 (1.26)	37.5 (3.62)	30.1 (3.15)
Light cluster	223	11.0 (4.16)	9	4.86 (0.43)	43.6 (1.20)	32.9 (2.88)	25.7 (2.10)
*Males*	*231*		*10*				
Heavy cluster	110	7.10 (2.12)	10	8.08 (0.64)	51.1 (1.41)	39.2 (3.48)	31.1 (2.55)
Light cluster	121	7.22 (1.89)	0	6.83 (0.40)	49.4 (1.24)	33.4 (2.55)	27.9 (1.71)
Total	626	9.33 (3.84)	68	6.10 (1.28)	46.2 (3.28)	35.1 (4.01)	28.2 (3.27)

Abbreviations: BMI, body-mass index; BW, body weight; CRL, crown-to-rump length; WC, waist circumference. All values are mean (sd) values for each individual across their lifespan from age 5 onward (e.g., means are the average of each adult's average weight across all individuals).

**Table 2:** Estimated heritability of obesity-related traits in the VRC (2000–2015)

*Trait*	*n*	*h*^*2*^	*P-value*	*c*^*2*^	*P-value*	*Sig. covariates*
*Adult measures*						
BW (kg)	626	**0.72 (0.07)**	**<0.0001**	0.05 (0.04)	0.0838	Sex, maternal diet, sex*maternal diet, ID(PN1), birth year
WC (cm)	347	**0.87 (0.09)**	**<0.0001**	**0.08 (0.05)**	**0.0471**	Sex
BMI	606	**0.65 (0.07)**	**<0.0001**	**0.06 (0.04)**	**0.0445**	Sex, maternal diet, sex*maternal diet, sex*ID(adult)
CRL (cm)	606	**0.65 (0.07)**	**<0.0001**	0.00 (0.00)	—	Sex, Maternal Diet, ID(PN1,PN2), Birth Year
Chronic obesity	626	**0.53 (0.51)**	**0.0351**	0.01 (0.18)	0.4821	Age, birth year, birth location
						
*Growth parameters*						
*BW (kg)*						
Asymptote	800	**0.61 (0.06)**	**<0.0001**	**0.05 (0.03)**	**0.0296**	Sex, maternal diet, sex*maternal diet, ID(PN1,PN2), sex*ID(PN1,PN2), birth year
Midpoint		**0.12 (0.05)**	**0.0003**	0.00 (0.00)	—	Sex, sex*maternal diet, ID(Adult), sex*ID(PN2,adult)
Rate		**0.23 (0.06)**	**<0.0001**	0.05 (0.04)	0.0629	Sex, maternal diet, sex*maternal diet, ID(PN1,adult), sex*ID(adult)
* CRL (cm)*						
Asymptote	800	**0.66 (0.06)**	**<0.0001**	0.02 (0.06)	0.2412	Sex, maternal diet, sex*maternal diet, ID(PN2), sex*ID(PN1,PN2), birth year
Midpoint		**0.13 (0.05)**	**0.0015**	0.01 (0.03)	0.4377	Sex, sex*ID(PN2)
Rate		**0.12 (0.07)**	**0.0131**	**0.07 (0.04)**	**0.0352**	Sex, sex*maternal diet, ID(PN1,PN2)

Abbreviations: BMI, body-mass index; BW, body weight; CRL, crown-to-rump length; WC, waist circumference. Emboldened text indicates statistical significance. Estimated variance components for narrow-sense heritability (h2) and maternal component of the environmental variance (c2) are presented with standard error in parentheses. Significant covariates are presented in this table only. All analyses (except WC, see Methods section) included sex, maternal diet during gestation, ID diet begun within the first 2 postnatal years (PN1), begun during the next 3 postnatal years (PN2), and during adulthood (adult), year of birth, and location of birth (UCLA or Wake Forest) as covariates. Adult measures were adjusted for mean age at time of measurements prior to analysis. Interaction effects are denoted by an asterisk (*) between interacting effects. Adult trait values other than Chronic Obesity are mean values for all adult measures for a given individual. Only adult individuals (ages 5 and above) were used for adult measures analysis. Growth parameters were derived from NLME models that included individuals aged from birth to adulthood that had at least 6 measuring points represented, excluding time points when females were pregnant. All values were inverse normalized prior to heritability assessments.

**Table 3:** Summary of linkage results for adult and growth phenotypes in the VRC

*Trait*	*Maximum LOD*	*Chr.*	*cM*	*Human associated region*	*Genes of interest in suggestive Loci*	*Associated human phenotypes*
*Adult measures*						
BW (kg)	1.77	13	54	—	—	—
WC (cm)	1.79	8	9	—	—	—
BMI	2.27	10	109	2q37.3	*RAMP1*,*FAM132B*,*HDAC4*,*CAPN10/GPR35*,*SNED1*,*PASK*,*HDLBP*,*STK25* and*TWIST2*	Type 2 diabetes, insulin dysregulation, coronary artery disease, thyroid disorder and lipid metabolism
CRL (cm)	1.89	5	15	16p13.1	*CLEC16A*,*SOCS1*,*TNP2*,*PRM3*, *RMI2*and*LITAF*	Obesity, Type 1 diabetes, insulin resistance and TNF-α pathway
	2.95	7	29	4q12–21.1	*CLOCK*,*TMEM165*,*EREG*,*CXCL11*,*STBD1*,*CXCL13*,*G3BP2*,*NUP54* and*SCARB2*	Obesity, BMI, abnormal adipose distribution, congenital disorder of glycosylation type Iik, glycogen metabolism, weight fluctuations, autoimmune thyroiditis, thymic hyperplasia and NF-κB pathway
	2.71	21	3	7p11.2	*VOPP1*,*LANCL2*,*EGFR* and*SEC61G*	Hypertension, insulinoma, atherosclerosis, hypercholesterolemia, thyroid dysfunction, insulin production and NF-κB pathway
						
*Growth Parameters*						
* BW (kg)*						
Asymptote	1.91	23	0		—	—
Midpoint	1.92	2	24	2q13.1	*MYBL2*,*IFT52*,*L3MBTL1/SGK2*,*SRSF6* and*PTPRT*	Type 2 diabetes, Type 1 diabetes
Rate	2.79	5	19	16p13.1	*CLEC16A*,*SOCS1*,*TNP2*,*PRM3*,*RMI2*,*LITAF*,*TXNDC11*,*TNFRSF17*,*CPPED1*,*PARN*,*PLA2G10*,*PDXDC1*,*NTAN1* and*PMM2*	Disorder of glycosylation type 1a, abnormal adipose distribution, Type 1 diabetes, obesity, hypertension, coronary artery disease, insulin resistance, lipid metabolism, TNF-α pathway and NF-κB pathway
	2.04	10	115	2q37.3	*CAPN10/GPR35*,*SNED1*,*PASK*,*HDLBP* and*STK25*	Insulin metabolism, Type 2 diabetes, coronary artery disease and pseudopseudohypoparathyroidism
	2.00	21	5	7p12.1	*POM121L12*	Glucose transport
	2.01	23	52	5q33.3	*FAXDC2*and*TIMD4*	Metabolic disorder, LDL/triglyceride levels and fatty acid biosynthesis
* CRL (cm)*						
Asymptote	2.11	7	29	4q21.1	*CXCL13*	Thymic hyperplasia
	2.26	21	0	7p11.2	*PHKG1*,*CCT6A*,*PSPH*,*VOPP1*,*LANCL2*,*EGFR* and*SEC61G*	Atherosclerosis, hypertension, glycogen storage, insulinoma, insulin dysregulation, early failure to thrive, TNF-α pathway and NF-κB pathway
Midpoint	1.62	10	17		—	—
Rate	1.64	4	8		—	—

Abbreviations: BMI, body-mass index; BW, body weight; CRL, crown-to-rump length; WC, waist circumference. All phenotypes represent age adjusted and inverse normal transformed values used in multipoint linkage analysis. Human associated region refers to the associated region on human chromosomes for genome-wide suggestive vervet QTL. Suggestive loci of interest are from all regions with genome-wide suggestive linkage, not only the area with the maximum LOD score. For a more complete list of loci with references, Supplementary Table 1.
