# Supplementary material for: Obesity and obesogenic growth are both highly heritable and modified by diet in a nonhuman primate model, the African green monkey (Chlorocebus aethiops sabaeus)
Source: Int J Obes (Lond). 2018 Feb 13;42(4):765–74. doi: 10.1038/ijo.2017.301 (PMC5984074; doi:10.1038/ijo.2017.301)
Supplement: Supplementary Figure Legends [file ijo2017301x1.docx]

**Supplemental Figure 1** All study individuals (n = 905) from the VRC used in these analyses (2000 – 2015). Each point represents a session in which BW measurements of that individual were collected (other measurements may or may not have been collected during those time). Point color indicates sex and whether or not that individual became chronically obese in their lifetime. The pink band represents the time points during which the VRC was fed the Intervention Diet. The dark gray band encompasses those individuals whose mothers were eating the Intervention Diet during their gestation. All points outside those bands were collected when the VRC was fed the Standard diet, and gestated while their mothers ate the Standard diet. Those individuals whose points are gray had undetermined chronic obesity status, but identifiable sex and enough measurements to be included in the cluster analysis.

**Supplemental Figure 2** Inter-site reliability in CRL measurements.

As mentioned in the Methods, inter-observer reliability was conducted at both UCLA and at Wake Forest, but was not conducted across sites. The measurements of crown-to-rump length (CRL) and waist circumference (WC) can be particularly sensitive to measurer bias. WC was not measured consistently between sites, so we only used WC data from Wake Forest in this manuscript. We have, however, used CRL measurements from both UCLA and Wake Forest. Matthew Jorgensen, current manager of the VRC at Wake Forest and a coauthor on this manuscript, conducted measurements of CRL at both sites, which suggests continuity. To test this assumption, we compared average adult CRL across sites.

This comparison was done by using only adult measures (age 5 and older), under the assumption that growth would have ceased by that age. To be consistent with methods in the manuscript, we also excluded all measurements on females taken when pregnant. We then separated measurements into two groups corresponding to measurement ‘Location’ (UCLA or Wake Forest) and averaged CRL measurements for each individual in each of the two groups. Inter-site reliability could not be directly assessed for CRL using paired average values from the same individual because there is not a single animal that was measured as an adult in UCLA who was also measured as an adult in Wake Forest for CRL. This may be an artifact of CRL being not as frequently collected while the VRC was at UCLA, and those animals that were measured in adulthood may have died or been sold prior to the colony transit to Wake Forest. As in the manuscript, we regressed mean age at measurement for each individual against CRL and used the residuals (‘CRLadj’) for the remainder of analyses to account for any potential remaining variation in CRL based on age. Finally, also as in the manuscript, we inverse normal transformed CRLadj (‘inCRLadj’) within both male and female samples. Instead of a paired approach, we constructed separate linear mixed models for both males and females with all significant fixed covariates from the adult analysis in our manuscript (categorical age at first exposure to ID) along with Location while using the relatedness matrix derived from the VRC pedigree as a random effect. Model output shows no significant effect of Location on inverse normalized mean adult CRLadj for either males (β = 0.4171, p = 0.67) or females (β = -0.0635, p = 0.90), suggesting that this measure has reasonable inter-site comparability. Based on these analyses, we feel confident that inter-site measurements of CRL are comparable for the purposes of our analyses.

**Supplemental Figure 3** Distribution of ages (represented by year of birth) in the adult-measures sample of vervets in the VRC.

**Supplemental Figure 4** Distribution of ages (represented by year of birth) in the growth sample of vervets in the VRC.

**Supplemental Figure 5** Mean and standard error of residual differences by sex in age-adjusted CRL for individuals fed a Standard diet (in black) and those who experienced a shift to ID (in grey) during (a) gestation (maternal shift to ID while gestating that individual), (b) during the first two years after birth (PN1), (c) during the subsequent three years after birth (PN2), and (d) during adulthood. Residual values were attained after regressing out significant covariates, here including age and growth cluster assignment.

**Supplemental Figure 6** Mean and standard error of residual differences by sex in age-adjusted WC for individuals fed a Standard diet (in black) and those who experienced a shift to ID (in grey) during (a) gestation (maternal shift to ID while gestating that individual), (b) during the first two years after birth (PN1), (c) during the subsequent three years after birth (PN2), and (d) during adulthood. Residual values were attained after regressing out significant covariates, here including age and growth cluster assignment.

**Supplemental Figure 7** Mean and standard error of residual differences by sex in *θ*_1,_ the asymptote of growth, for BW for individuals fed a Standard diet (in black) and those who experienced a shift to ID (in grey) during (a) gestation (maternal shift to ID while gestating that individual), (b) during the first two years after birth (PN1), (c) during the subsequent three years after birth (PN2), and (d) during adulthood. Residual values were attained after regressing out significant covariates.

**Supplemental Figure 8** Mean and standard error of residual differences by sex in -*θ*_2_/*θ*_3_, the midpoint of growth, for BW for individuals fed a Standard diet (in black) and those who experienced a shift to ID (in grey) during (a) gestation (maternal shift to ID while gestating that individual), (b) during the first two years after birth (PN1), (c) during the subsequent three years after birth (PN2), and (d) during adulthood. Residual values were attained after regressing out significant covariates.

**Supplemental Figure 9** Mean and standard error of residual differences by sex in *θ*_3,_ the growth rate constant, for BW for individuals fed a Standard diet (in black) and those who experienced a shift to ID (in grey) during (a) gestation (maternal shift to ID while gestating that individual), (b) during the first two years after birth (PN1), (c) during the subsequent three years after birth (PN2), and (d) during adulthood. Residual values were attained after regressing out significant covariates.

**Supplemental Figure 10** Mean and standard error of residual differences by sex in *θ*_1,_ the asymptote of growth, for CRL for individuals fed a Standard diet (in black) and those who experienced a shift to ID (in grey) during (a) gestation (maternal shift to ID while gestating that individual), (b) during the first two years after birth (PN1), (c) during the subsequent three years after birth (PN2), and (d) during adulthood. Residual values were attained after regressing out significant covariates.

**Supplemental Figure 11** Mean and standard error of residual differences by sex in -*θ*_2_/*θ*_3_, the midpoint of growth, for CRL for individuals fed a Standard diet (in black) and those who experienced a shift to ID (in grey) during (a) gestation (maternal shift to ID while gestating that individual), (b) during the first two years after birth (PN1), (c) during the subsequent three years after birth (PN2), and (d) during adulthood. Residual values were attained after regressing out significant covariates.

**Supplemental Figure 12** Mean and standard error of residual differences by sex in *θ*_3,_ the growth rate constant, for CRL for individuals fed a Standard diet (in black) and those who experienced a shift to ID (in grey) during (a) gestation (maternal shift to ID while gestating that individual), (b) during the first two years after birth (PN1), (c) during the subsequent three years after birth (PN2), and (d) during adulthood. Residual values were attained after regressing out significant covariates.
