# Supplementary figures and images for: Obesity and obesogenic growth are both highly heritable and modified by diet in a nonhuman primate model, the African green monkey (Chlorocebus aethiops sabaeus)
Source: Int J Obes (Lond). 2018 Feb 13;42(4):765–74. doi: 10.1038/ijo.2017.301 (PMC5984074; doi:10.1038/ijo.2017.301)

Female

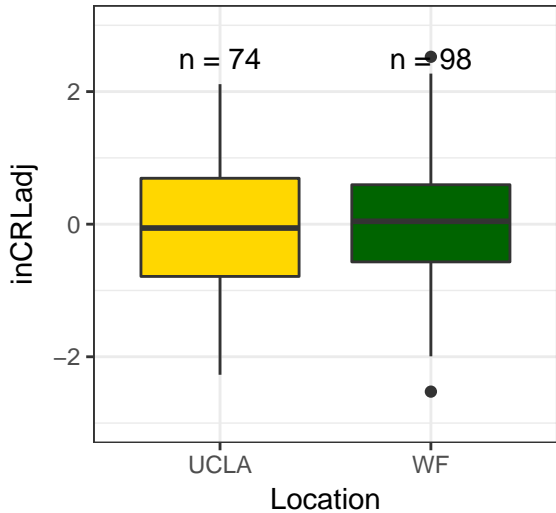

Male

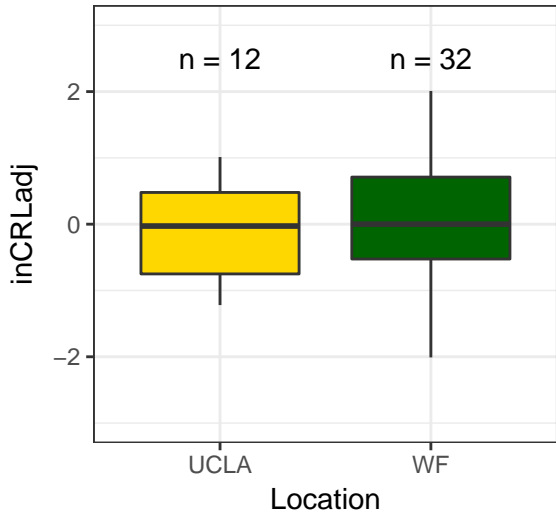

Supplement: Supplementary Figure 2 [file ijo2017301x4.pdf]

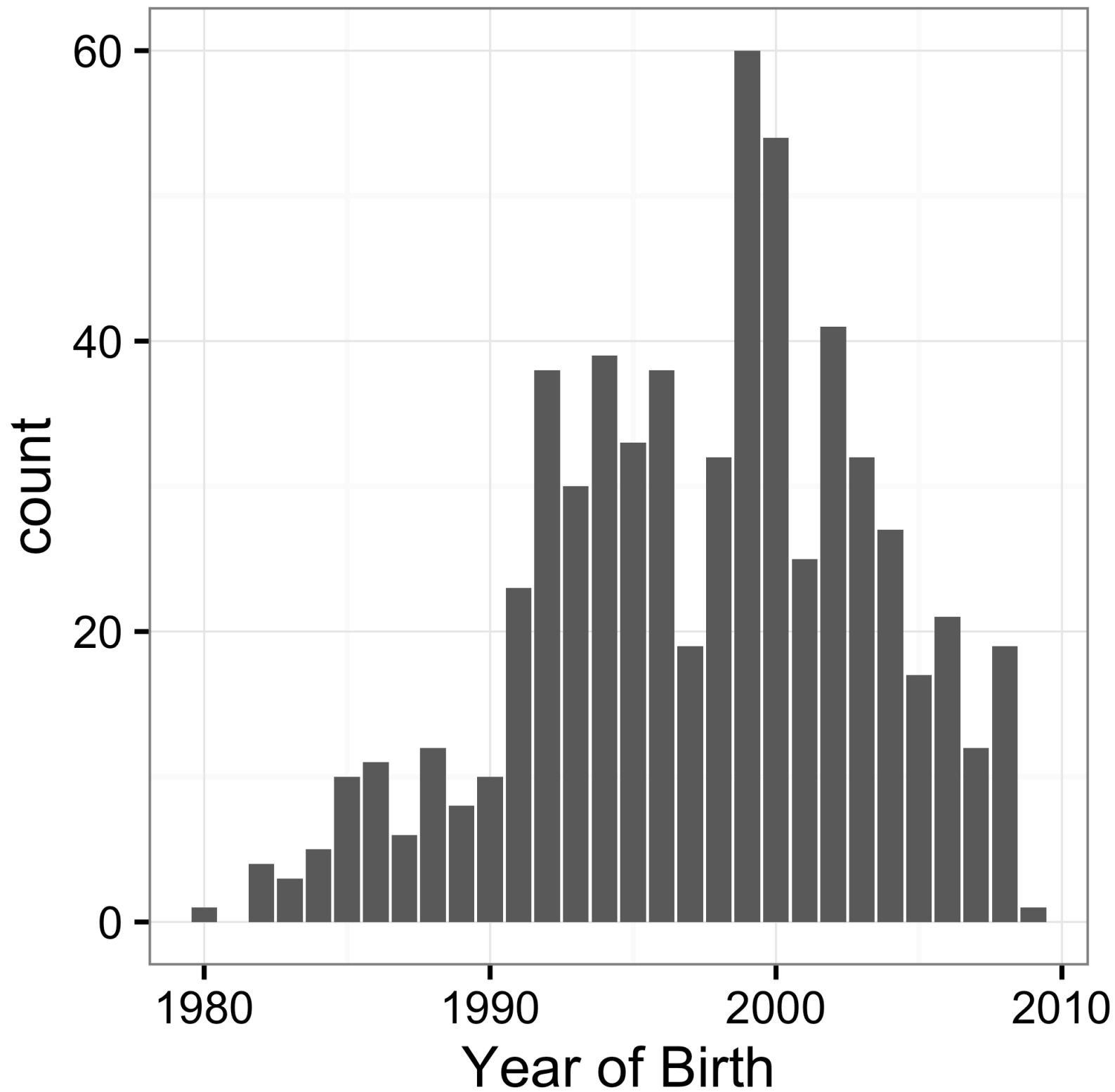

Supplement: Supplementary Figure 3 [file ijo2017301x5.pdf]

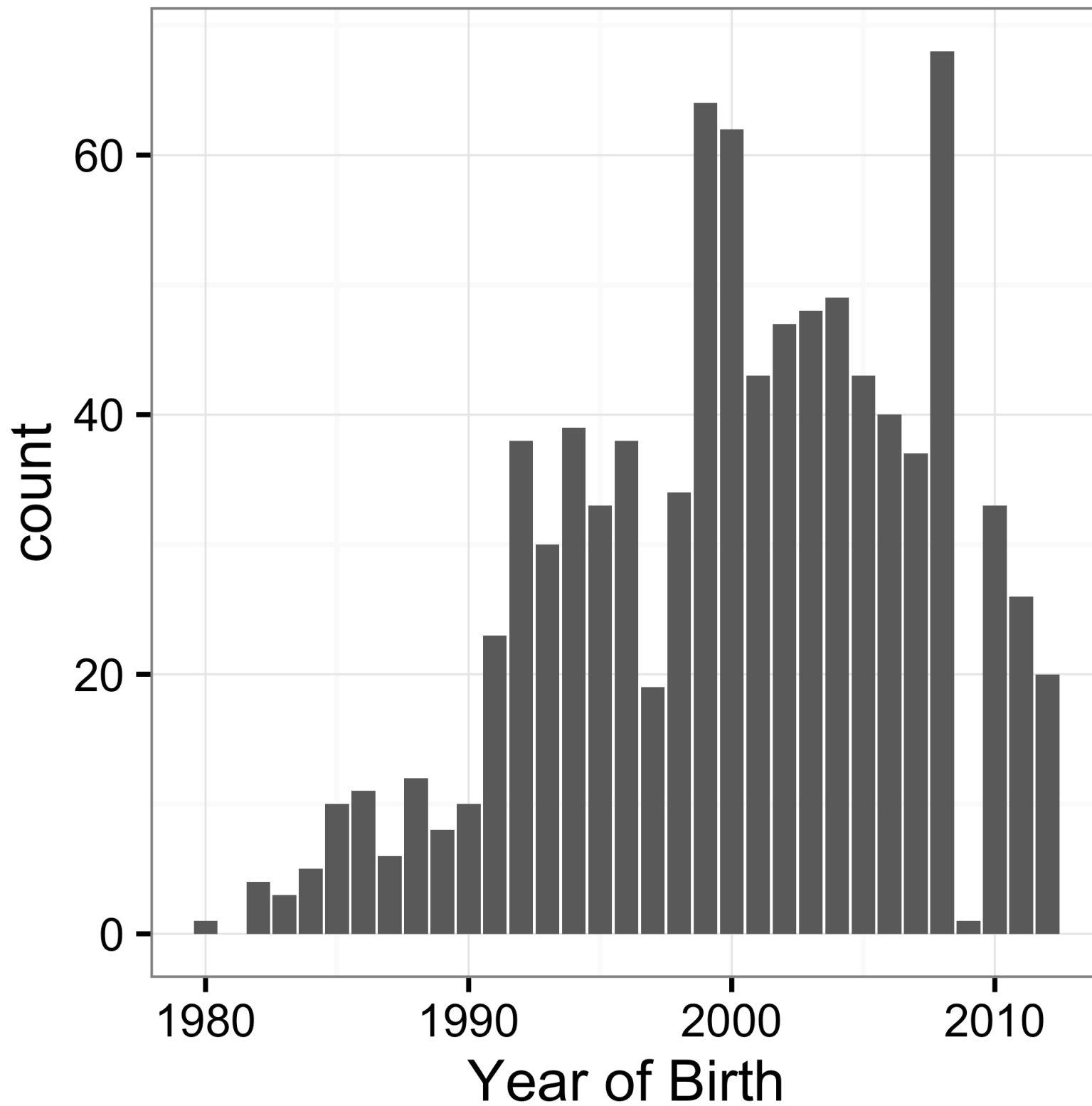

Supplement: Supplementary Figure 4 [file ijo2017301x6.pdf]

Mean Residual Adjusted CRL

**a) Gestation**

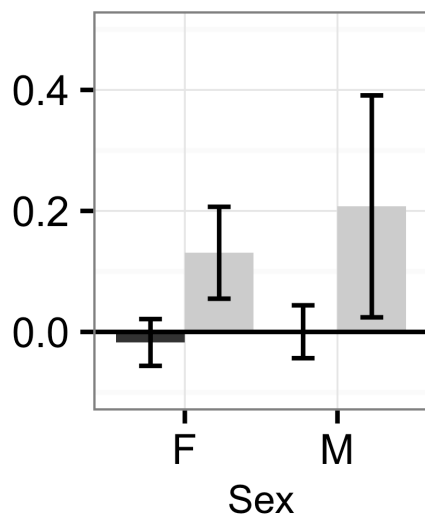

**b) PN1**

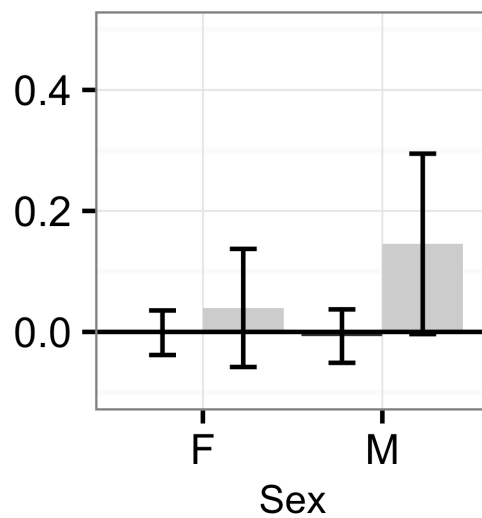

**c) PN2**

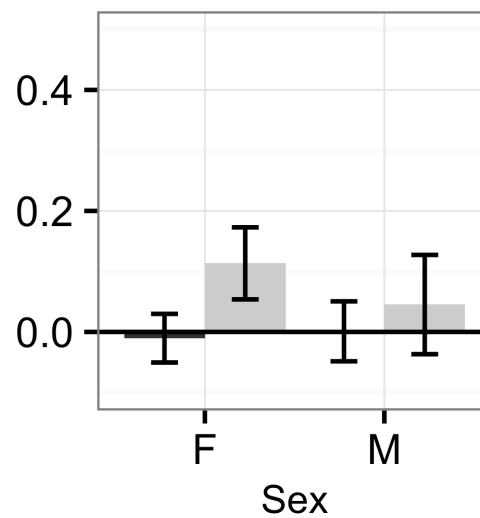

**d) Adulthood**

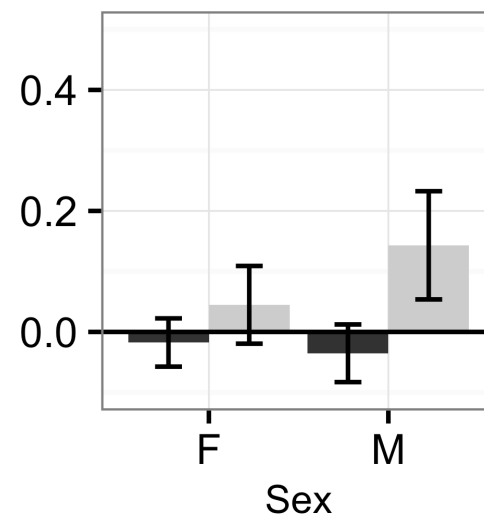

Supplement: Supplementary Figure 5 [file ijo2017301x7.pdf]

Mean Residual Adjusted WC

**a) Gestation**

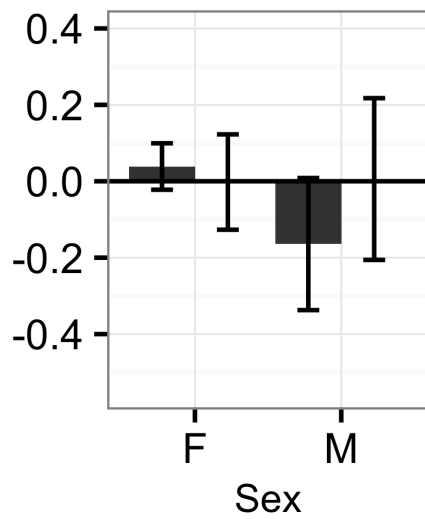

**b) PN1**

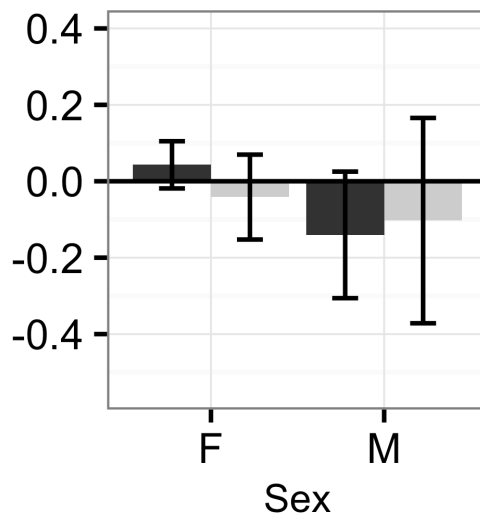

**c) PN2**

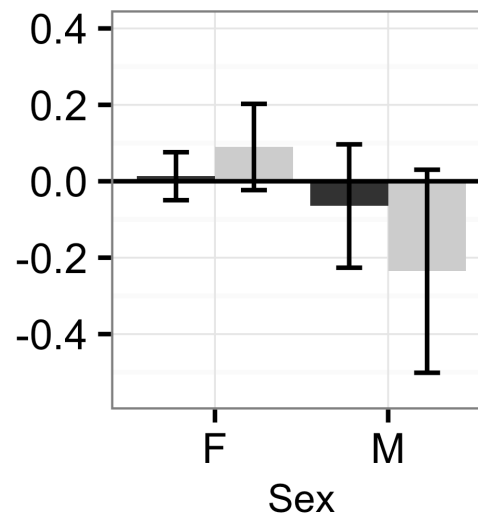

**d) Adulthood**

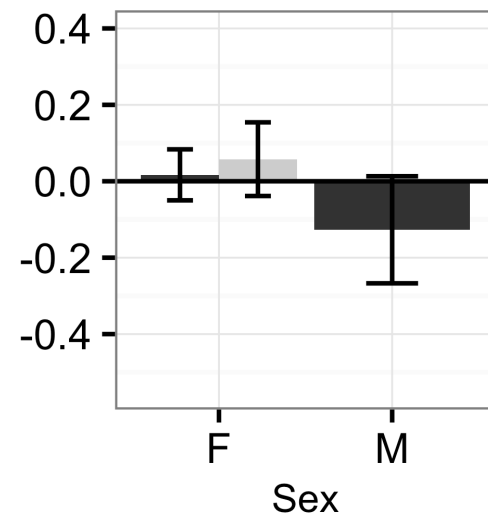

Supplement: Supplementary Figure 6 [file ijo2017301x8.pdf]

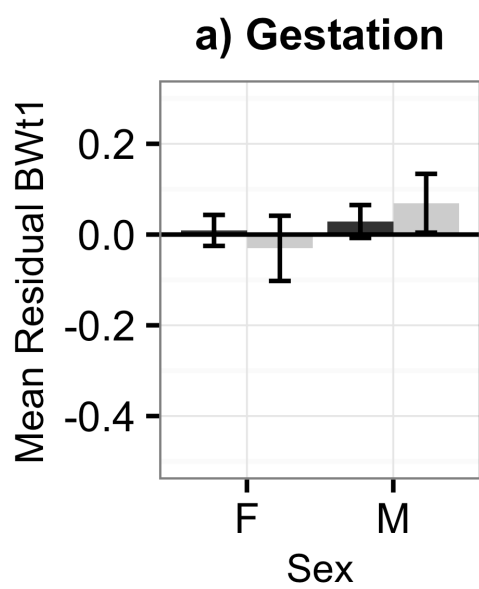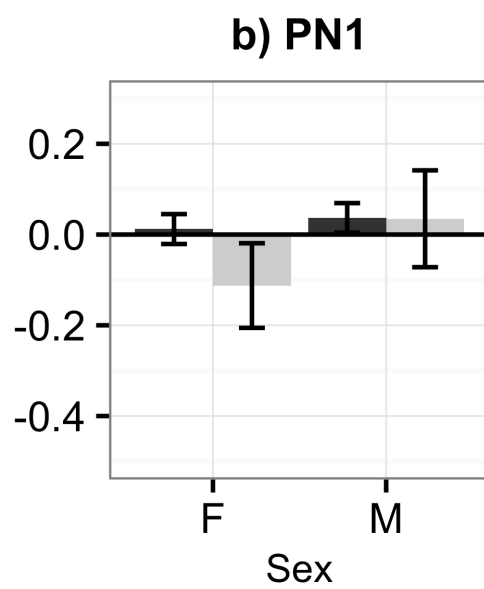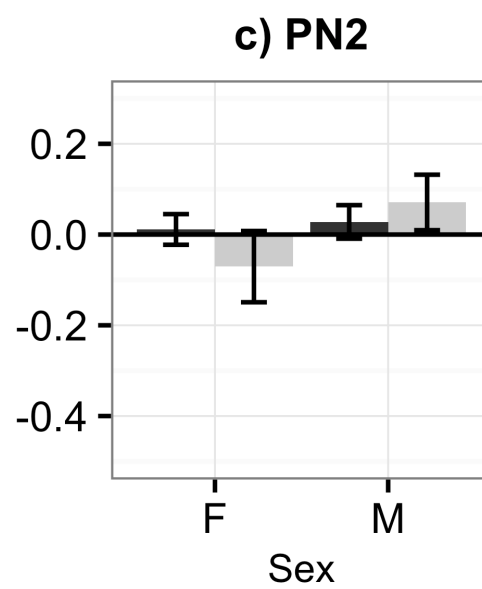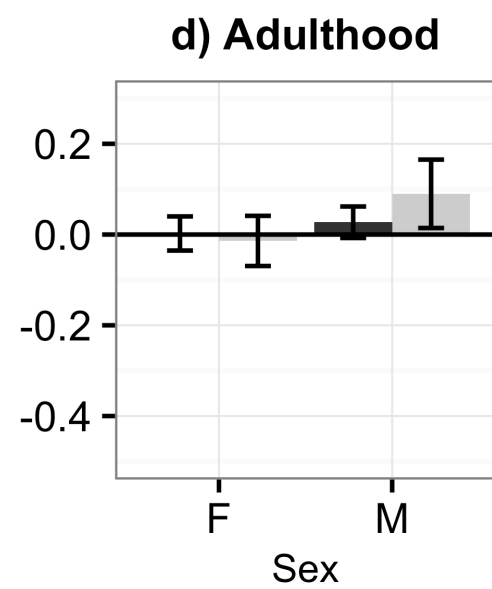

Supplement: Supplementary Figure 7 [file ijo2017301x9.pdf]

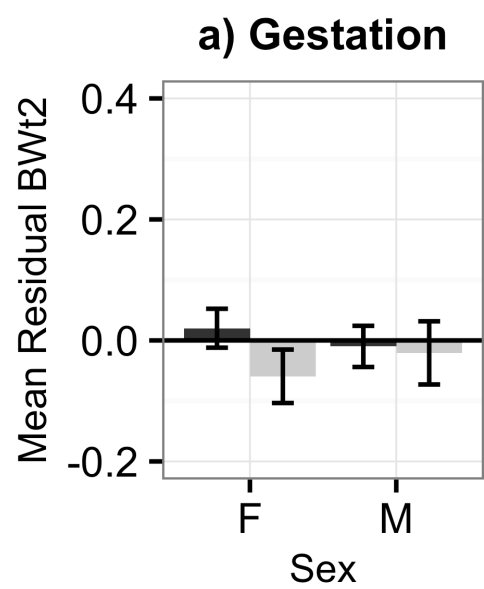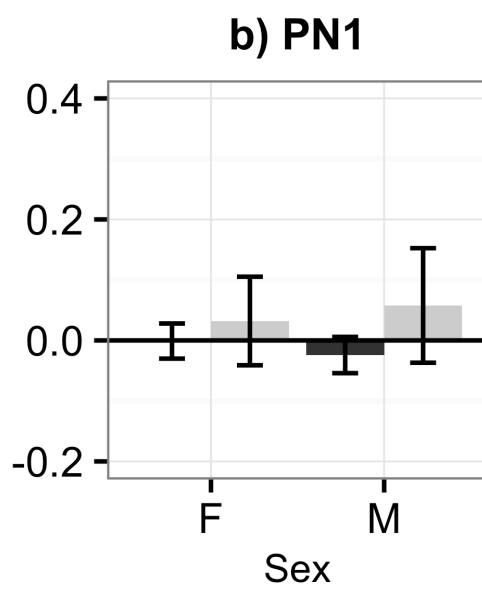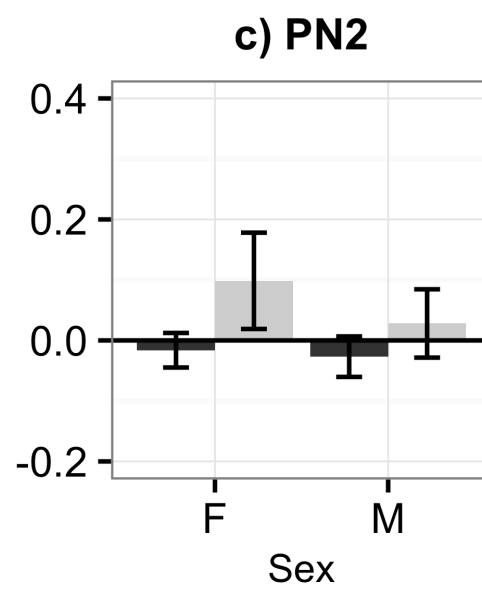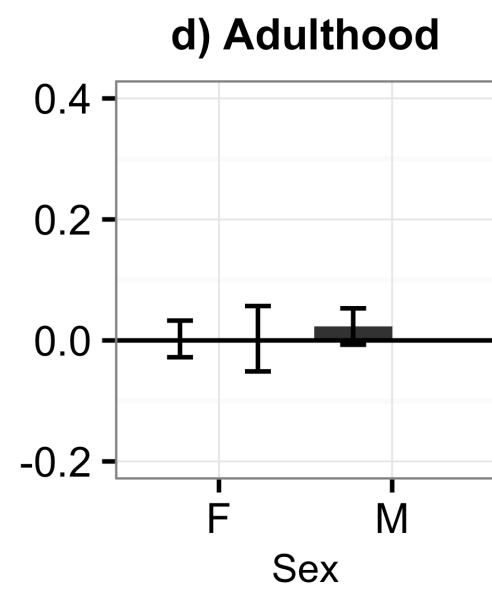

Supplement: Supplementary Figure 8 [file ijo2017301x10.pdf]

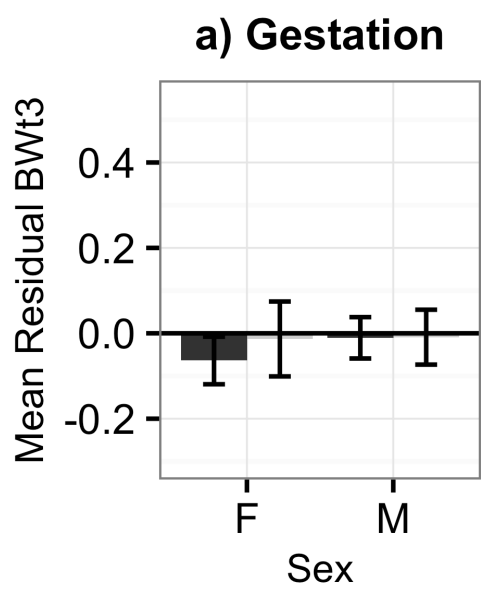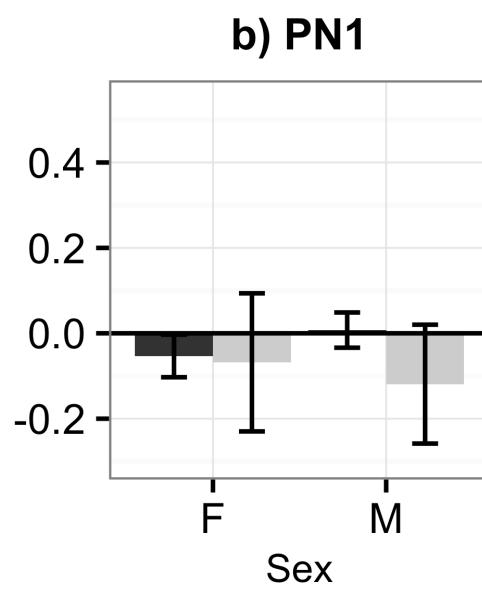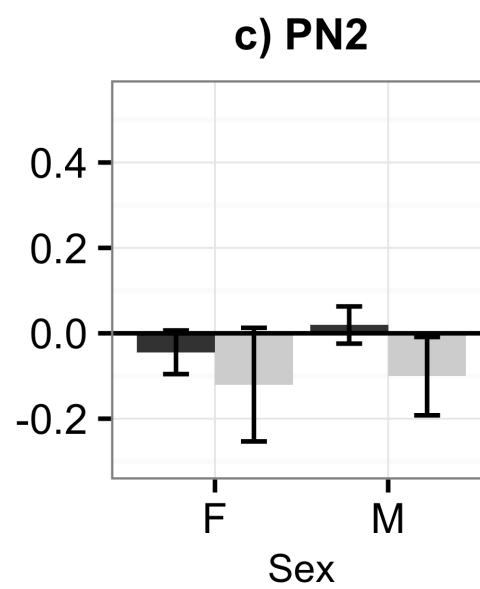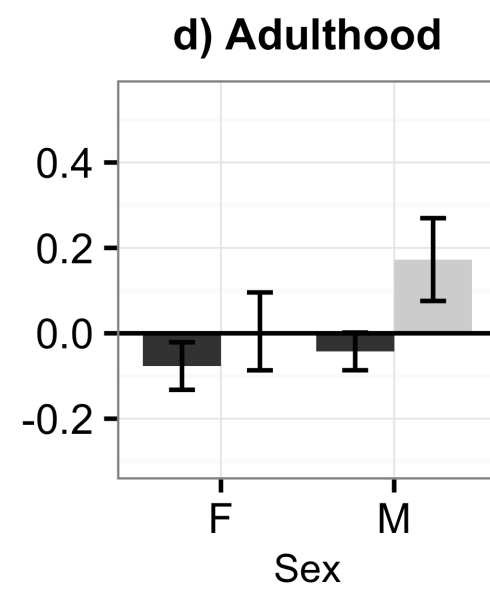

Supplement: Supplementary Figure 9 [file ijo2017301x11.pdf]

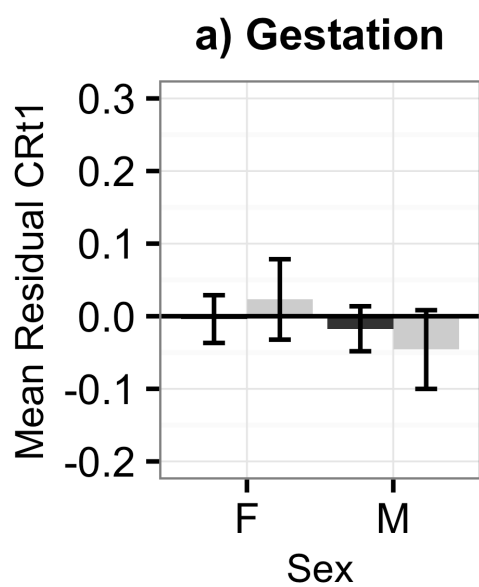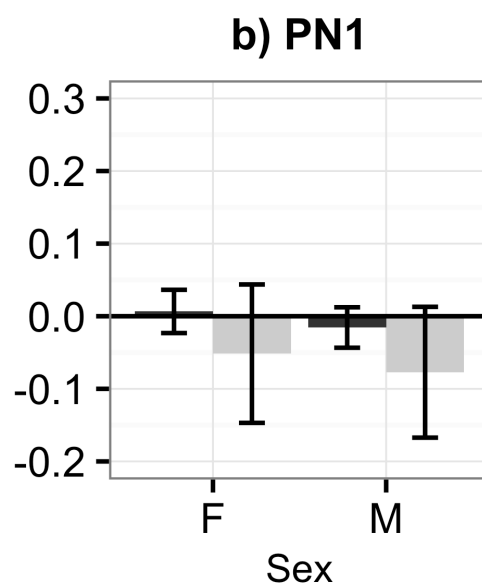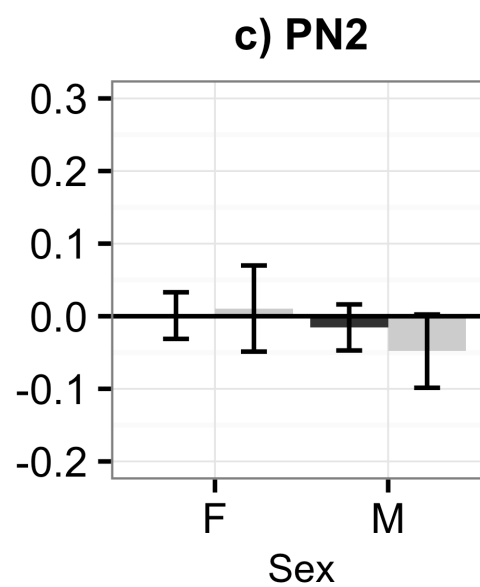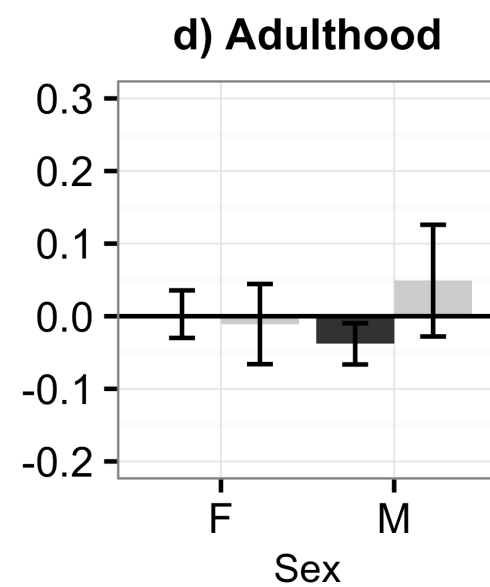

Supplement: Supplementary Figure 10 [file ijo2017301x12.pdf]

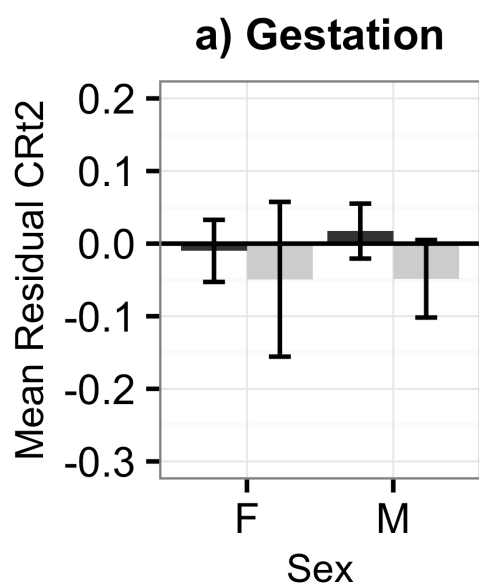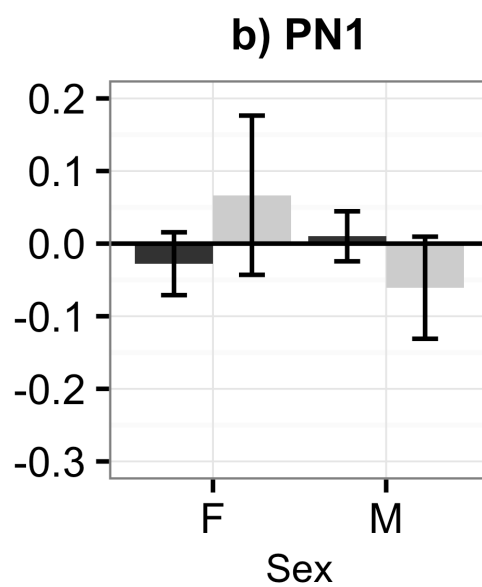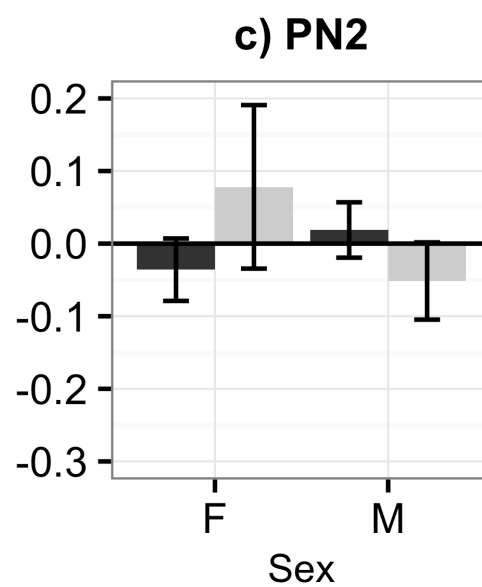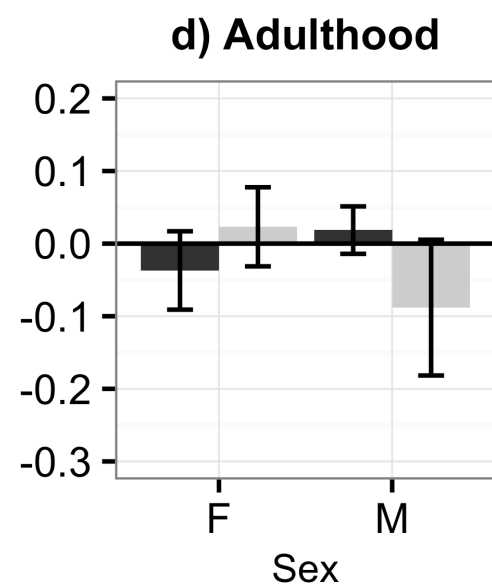

Supplement: Supplementary Figure 11 [file ijo2017301x13.pdf]

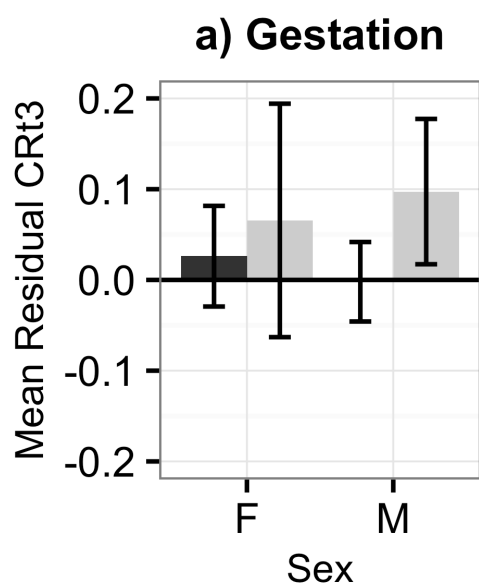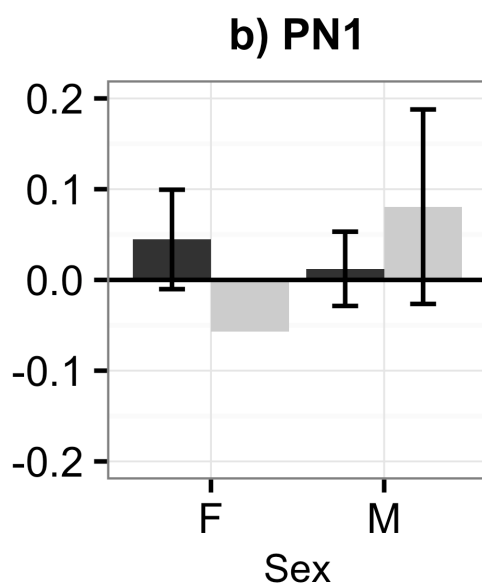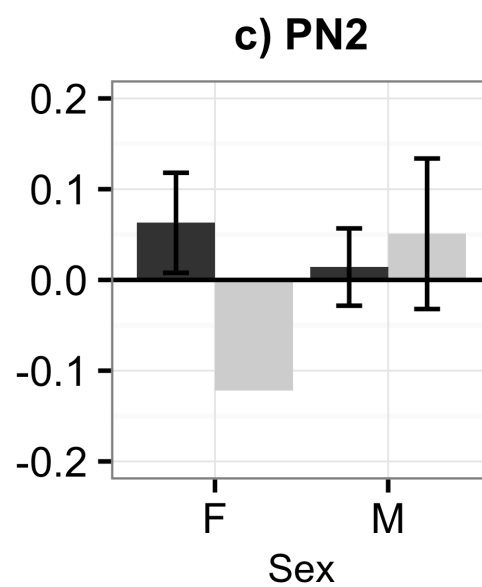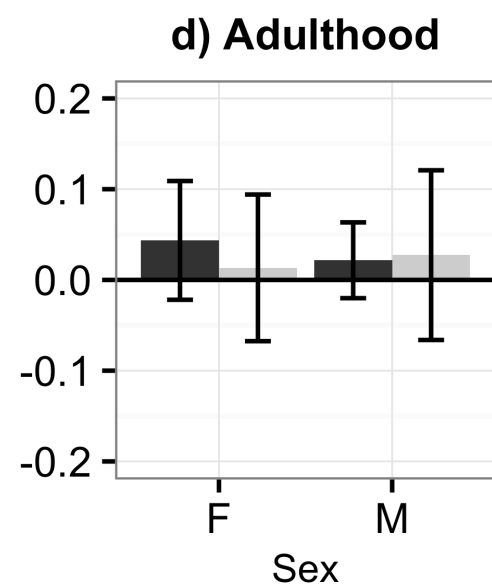

Supplement: Supplementary Figure 12 [file ijo2017301x14.pdf]
