# Supplementary material for: Obesity and obesogenic growth are both highly heritable and modified by diet in a nonhuman primate model, the African green monkey (Chlorocebus aethiops sabaeus)
Source: Int J Obes (Lond). 2018 Feb 13;42(4):765–74. doi: 10.1038/ijo.2017.301 (PMC5984074; doi:10.1038/ijo.2017.301)

VRC Growth & Adult Dataset, 2000 − 2015

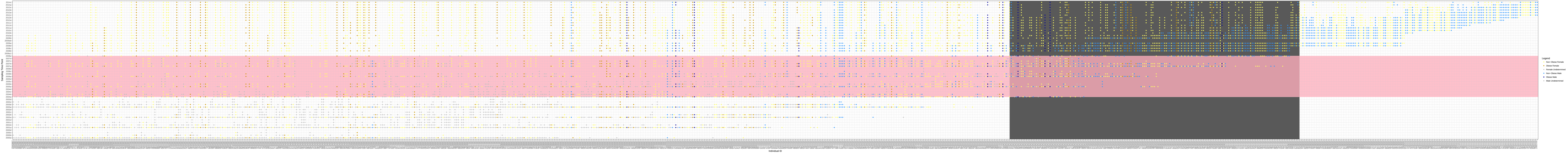

Supplement: Supplementary Figure 1 [file ijo2017301x3.pdf]
